# Harbour porpoises react to low levels of high frequency vessel noise

**DOI:** 10.1038/srep11083

**Published:** 2015-06-22

**Authors:** Monika Dyndo, Danuta Maria Wiśniewska, Laia Rojano-Doñate, Peter Teglberg Madsen

**Affiliations:** 1 Zoophysiology, Department of Bioscience, Aarhus University, C. F. Moellers Alle 3, 8000 Aarhus C, Denmark; 2 Marine Mammal Research, Department of Bioscience, Aarhus University, Frederiksborgvej 399, 4000 Roskilde, Denmark; 3 Murdoch University Cetacean Research Unit, School of Veterinary and Life Sciences, Murdoch University, South Street, Murdoch, Western Australia 6150, Australia

## Abstract

Cetaceans rely critically on sound for navigation, foraging and communication and are therefore potentially affected by increasing noise levels from human activities at sea. Shipping is the main contributor of anthropogenic noise underwater, but studies of shipping noise effects have primarily considered baleen whales due to their good hearing at low frequencies, where ships produce most noise power. Conversely, the possible effects of vessel noise on small toothed whales have been largely ignored due to their poor low-frequency hearing. Prompted by recent findings of energy at medium- to high-frequencies in vessel noise, we conducted an exposure study where the behaviour of four porpoises (*Phocoena phocoena*) in a net-pen was logged while they were exposed to 133 vessel passages. Using a multivariate generalised linear mixed-effects model, we show that low levels of high frequency components in vessel noise elicit strong, stereotyped behavioural responses in porpoises. Such low levels will routinely be experienced by porpoises in the wild at ranges of more than 1000 meters from vessels, suggesting that vessel noise is a, so far, largely overlooked, but substantial source of disturbance in shallow water areas with high densities of both porpoises and vessels.

High efficiency of underwater propagation of sound^1^ makes it of particular importance to many marine animals, including cetaceans. Auditory scene analysis allows them to navigate and localise possible threats, and acoustic communication facilitates social interactions, individual or group recognition, courtship behaviour, or potential group actions such as foraging^2^,^3^. Toothed whales are particularly dependent on sound due to their reliance on echolocation for prey detection, localisation and discrimination, as well as for orientation^4^,^5^,^6^. The dramatic increase in human activities and encroachment at sea in the last century has led to a substantial increase in ambient noise levels^7^,^8^ that may have the potential to negatively affect the auditory scene analysis, behaviour, and physiology of cetaceans with broad scale implications for the fitness of individuals and populations^9^,^10^.

Recently, a number of studies have focused on high-power transient noise sources such as sonars [e.g. 11,12], airguns [e.g. 13,14] and pile driving [e.g. 15]. Yet, shipping is by far the most dominant anthropogenic source of underwater noise at low frequencies, and is responsible for the vast majority of anthropogenic noise inputs to the marine environment^16^,^17^. Some baleen whales exploit similar frequency bands to the frequencies of peak power outputs from large vessels in deep water^18^ and are therefore generally considered being at the highest risk for adverse effects of ship noise^19^,^20^.

Toothed whales are not normally considered when evaluating the impacts of ship noise due to their poor hearing at frequencies below 1 kHz^3^,^4^. The sensitivity of their hearing gradually improves with increasing frequency and reaches its best between 10 and 120 kHz (<55 dB re 1 μPa)^3^,^21^. Perhaps surprisingly, given their hearing abilities, several studies have demonstrated that harbour porpoises do show what appears to be avoidance behaviour in response to vessels at long ranges^22^,^23^, where the radiated noise, rather than the physical presence of the vessel, is more likely to deliver the negative stimulus. Many small toothed whale species inhabit shallow waters which are high productivity areas^24^ that have some of the heaviest vessel traffic densities of any marine habitats^17^. However, shallow water environment acts as a steep high-pass filter were the low-frequency sounds do not propagate well^25^. Therefore this, in combination with the poor low-frequency hearing of porpoises, suggests that porpoises may respond to noise energy at mid- or high-frequencies that are present in vessel noise^26^,^27^, but currently not considered when estimating noise impact on cetaceans^28^,^29^.

Here, we test this hypothesis by studying the behaviour of captive harbour porpoises in a net pen being exposed to noise of passing vessels. We show that a strong, stereotyped, behavioural response in the form of porpoising is triggered by low levels of high-frequency component of vessel noise that can occur at more than 1000 meters from the source. The implication is that thousands of porpoises in shallow water habitats with dense vessel traffic may potentially face daily, repeated noise-induced behavioural disruptions, which is a potentially large, but so far, overlooked conservation issue.

## Results

Vessel noise from 133 boats was recorded at two stations across the net pen a total of 225 times during the study period, along with observations of porpoise behaviour. Implementation of a set of selection criteria (see Methods) reduced the total number of good quality recordings to 80 (14 registered at the left station and 66 at the right station). The selected recordings included noise from vessels of various size and design: from sailing boats moving on engine, through 4-10 m recreational boats with outboard engines, to fishing boats up to tens of meters long with inboard engines, and a single military vessel. In 22 cases (27.5%), a very robust and stereotypical reaction, in the form of porpoising (Supplementary Vid. S1), was observed when different boats were passing the net-pen complex.

### Echosounder pulses

First, we tested if the reactions were in fact triggered by vessel noise and not echosounders. Among 80 recorded vessels, 31 (39%) had a high-frequency (200 kHz) echosounder turned on (no other echosounders were recorded). A distinct reaction of the porpoises was observed in the presence of 11 of them (35%), but no statistically significant relationship between the presence of echosounder pulses and reaction was shown (p-value_BHY_ = 0.9464; Supplementary Tab. S1). The effect of the cumulative sound exposure levels (cSELs) of the echosounder signals was then examined to test whether the summed exposure level of several transients at 200 kHz could explain the initiation of porpoising (Supplementary Fig. S1). The cSELs in 30-second-long time windows with most energy reached values between 105–145 dB re 1 μPa^2^s. The commencement of porpoising did not coincide with the largest changes in the cSEL, nor a particular cSEL value (Supplementary Fig. S1) and there was no significant difference between the cSELs of echosounder pulses from vessels that did, and did not, elicit the response (p-value_BHY_ = 0.8170; Supplementary Tab. S1). The high-frequency echosounders were therefore unlikely to have caused the observed porpoising reactions, which suggest that the vessel noise itself triggered the responses.

### Broadband root-mean-square sound pressure level

**Figure 1:**
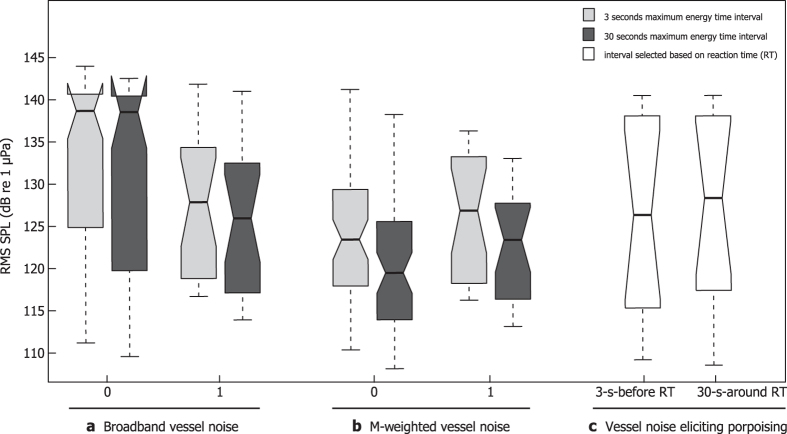
The distribution of rms sound pressure level calculated over different time intervals. (**a**) 3 seconds and 30 seconds of broadband vessel noise with maximum energy, (**b**) 3 seconds and 30 seconds of M-weighted vessel noise with maximum energy, (**c**) 3 seconds before and 30 seconds around reaction time (RT) - only for vessel noise eliciting porpoising behaviour. The thick line inside the box shows the median; the lower and upper edges of the box indicate the 1st and 3rd quartile, respectively; whiskers bound the minimum and maximum of the distributions. 0 - no reaction, 1 - reaction (porpoising) was observed. rms = root-mean-square.

The root-mean-square (rms) measure of sound pressure critically depends on the length of the time window over which the squared pressures are averaged^30^. Here, sliding time windows containing noise from passing vessels were moved so as to contain maximum energy in a 3-second- and 30-second-window. Additionally, segments of 3 seconds before and 30 seconds around the time of porpoise reaction were selected. The differences in rms pressure level between averaging windows of different durations and positions with respect to noise energy and time of reaction were negligible (Fig. 1a,c) and statistically insignificant (p = 0.7869). Therefore, a 30-second-long averaging window was used for all further analyses.

We proceeded to test if broadband rms received levels could explain the reactions as suggested by Southall *et al.*^19^ in their noise exposure criteria. Counter to this prediction, the broadband rms level was higher when porpoises showed no reaction to vessel noise (Fig. 1a). Results of a generalised linear mixed-effects model (GLMM) corroborated this finding by demonstrating that the association between the broadband rms sound pressure level and probability of reaction was not statistically significant (p-value_BHY_ = 0.8414; Supplementary Tab. S1). This suggests that certain spectral components, rather than the overall received level, would trigger the response.

### Spectral characteristics of the vessel noise

**Figure 2:**
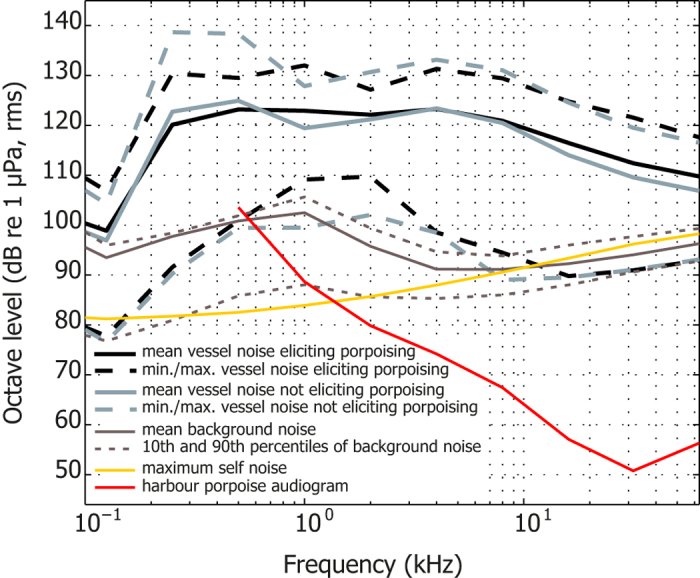
Mean, minimum and maximum of vessel noise (30 seconds with maximum energy) shown in octave bands superimposed on a harbour porpoise audiogram [red line; (adapted from 31)]. Noise evoking porpoising behaviour is indicated in black. Mean octave levels (solid) and 10^th^ and 90^th^ percentiles (dotted) of background noise are shown as grey lines. The maximum self-noise of the recording system is indicated by the yellow solid line.

**Figure 3:**
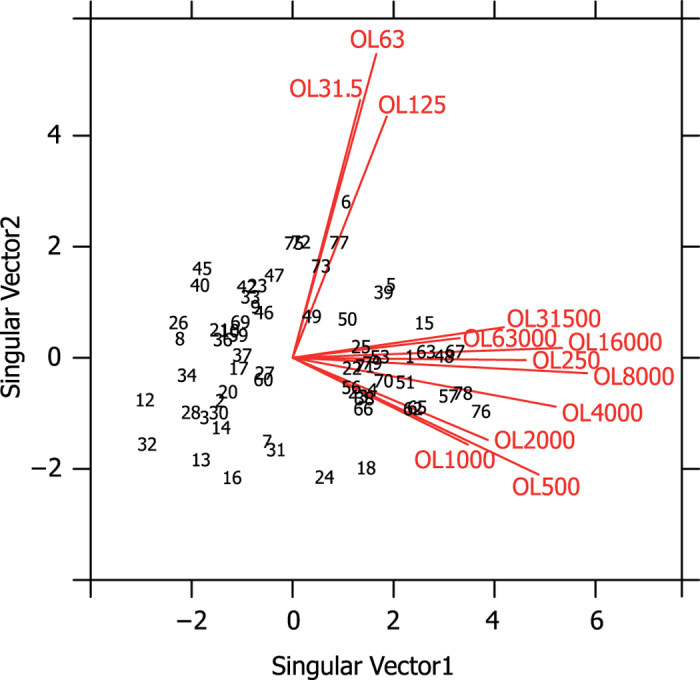
Biplot^32^ representing the correlation structure of the dataset in a two-dimensional space (see more details about this analysis in Methods section). In the biplot, black numbers represent the observations and the red vectors represent the variables. Axes refer to the first two singular vectors of the singular value decomposition. Observation scores are in deviation from their average for each of these singular vectors (the values were centred by variable). Note the homogeneous distribution of observations with no groups or extreme values and the clear aggregation of vectors into two different groups: low-frequency bands (OL31.5, OL63 and OL125 Hz), and high-frequency bands (OL250–OL63000 Hz). OL = octave level.

Power spectral density analysis of the vessel noise showed that it was broadband with most power at frequencies below 10 kHz (Supplementary Fig. S2). On average, the octave levels at frequencies greater than 500 Hz were between 20 and 60 dB above the porpoise audiogram (Fig. 2). Levels below 250 Hz were likely below the hearing threshold of porpoises^21^,^31^. To identify the frequency components of the vessel noise that were most likely to cause the behavioural response of the porpoises, a GLMM was performed for each of the 12 octave bands with centre frequencies between 31.5 Hz and 63 kHz. Additionally, we performed two more GLMMs using third-octave bands with centre frequencies at 63 and 125 Hz proposed by the Marine Strategy Framework Directive (MSFD) as indicators of general noise levels from continuous sources such as boats^29^. The mean, standard deviation (SD), median and interquartile ranges for all variables included in the GLMMs are shown in Supplementary Tab. S2. The results showed a statistically non-significant relationship between the porpoise reaction and both the 63- and 125-Hz third-octave bands (p-value_BHY_ = 0.8414 and 1.0000, respectively; Supplementary Tab. S1). In contrast, results of the GLMMs for the octave bands indicated a statistically significant, positive association between the probability of porpoising and rms levels in bands with centre frequencies at 500, 2000, 16000 and 31500 Hz (p-value_BHY_ = 0.0276, 0.0348, 0.0331, 0.0331, respectively; see odd ratios (OR) and p-values for all variables in Supplementary Tab. S1). Moreover, a two-dimensional biplot (see Methods and 32), representing 78% of the variance of the data, revealed a homogeneous display of observation points with no groups or extreme values. However, the biplot indicated two clear groups of vectors representing the octave bands with centre frequencies between 31.5 and 125 Hz and, separately, from 0.25 to 63 kHz (Fig. 3). Based on these findings, the correlated bands were merged into broader bandwidths, low- (31.5–125 Hz) and high-frequency (0.25–63 kHz), and their effects on porpoise reaction were tested. The results showed a statistically non-significant relationship between porpoise probability of reaction and sound pressure level at low-frequencies (p-value_BHY_ = 0.8414; Supplementary Tab. S1). However, a statistically significant impact of sound at high frequencies on porpoise behaviour was detected, indicating that higher levels of noise at these frequencies lead to an increase in the probability of reaction [OR = 1.37 (95% CI 1.09–1.73), p-value_BHY _= 0.0273; Supplementary Tab. S1].

The prevalence of high-frequency bands as explanatory variables prompted us to test if the M-weighting proposed by Southall *et al.*^19^ could be used as a simple response variable for practical implementation. The idea appears logical in view of the fact that the rms sound pressure level computed over a high-pass-filtered version of the vessel noise to some degree matches the high frequency hearing of porpoises^31^. A box plot was created to examine the distributions of the rms pressure levels of high-frequency M-weighted (cut-off frequencies: 200 Hz - 100 kHz;^19^) noise that did and did not elicit a behavioural response (Fig. 1b). Compared to the non-weighted data (Fig. 1a), a clear change in the level distributions was observed, with a higher level of M-weighted noise coinciding with a higher probability of porpoise response. This observation was supported by the GLMM results [OR = 1.41 (95% CI 1.12–1.78), p-value_BHY_ = 0.0273; Supplementary Tab. S1].

## Discussion

The most common and dominant contributors of anthropogenic noise in water are ships that radiate noise continuously at high levels^16^,^17^. Despite this, very little attention has been given to the effects of ship noise on small toothed whales that often inhabit waters with considerable vessel activity^33^. An argument for dismissing effects of vessel noise on small toothed whales is their poor hearing at low frequencies^31^ where large vessels radiate the most noise power^7^. Nevertheless, porpoises have been shown to avoid vessels at substantial ranges suggesting that they may in fact respond to low levels of vessel noise^22^,^23^. To test that hypothesis, we here used a stereotyped behavioural response as a measure of behavioural impact^34^ of a large number of vessel passes recorded with broadband, calibrated hydrophones. We show that despite long-term residence in a harbour, an environment that is inseparable from man-made noise, the four porpoises reacted in a manner of porpoising in the presence of almost 30% of boats where noise was recorded, lending little support for habituation effects.

Current recommendations of continuous underwater noise exposure criteria often stipulate a certain broadband rms level that cannot be exceeded^19^. Our data imply (Fig. 1a; Supplementary Tab. S1) that broadband rms levels cannot be used to predict behavioural responses to vessel noise of harbour porpoises, a high-frequency species^31^. This, in turn, suggests that exposure levels in certain spectral bands may be responsible for the observed responses.

An example of specific spectral bands proposed for quantifying the impact of vessel noise on marine life is that of the European MSFD recommending that levels in third-octave bands centred around 63 and 125 Hz serve as indicators of good environmental status^28^,^29^. However, we demonstrate that received levels in these two low-frequency bands cannot explain the observed behavioural responses, and the biplot analysis (Fig. 3) reveals a very weak association between the low- (31.5–125 Hz) and the medium- to high-frequency (0.25–63 kHz) octave bands of the noise where porpoise hearing is much better^31^. The latter finding is consistent with recent recordings of larger vessels in shallow water^27^. The proposed 63- and 125-Hz bands of the MSFD are therefore unsuited for establishing exposure limits for behavioural effects of vessel noise on porpoises and likely also for other small toothed whales, and they are in general poor proxies for noise loads at higher frequencies in shallow water environments^27^.

Rather, we show that higher levels of medium- to high-frequency components (0.25–63 kHz octave bands) of vessel noise significantly increase the probability of porpoising (Supplementary Tab. S1; Fig. 2). Thus, the porpoises responded to increases in the part of the noise spectrum where their hearing is good^21^,^31^, implying that the onset of a behavioural response is triggered by the perceived loudness of the sound^35^,^36^. This finding lends weight to the recent proposal by Tougaard *et al.*^37^ that behavioural responses of porpoises can be predicted from a certain level above their threshold at any given frequency. However, the extent to which noise affects an animal’s behaviour may also be determined by the background noise^38^. Our results suggest that behavioural and environmental covariates do affect the response threshold level of harbour porpoises, as the mean onset level of 123 dB re 1 μPa (rms, M-weighted; ranging from 113 to 133 dB re 1 μPa) for the porpoising behaviour is only slightly above the levels of noise that did not trigger the reaction (120 dB re 1 μPa, rms, M-weighted; ranging from 108 to 138 dB re 1 μPa). Nevertheless, such low levels are routinely encountered by porpoises in the wild from passing vessels at ranges of more than 1 km^27^,^39^ which can then explain the reported vessel avoidance of porpoises at considerable ranges^22^,^23^. Consequently, if wild porpoises respond to the same levels as documented here^34^, vessel noise may in heavily trafficked areas have a large, but so far undetected, effect on porpoises and potentially also on other small toothed whales^40^,^41^.

Porpoising and other behavioural responses to ship noise may be short-term, but they come at the cost of the energetic investment in moving, lost opportunities in foraging and social behaviour, as well as potential abandonment of calves. Thus, repeated vessel-noise-induced short term behavioural disruptions, as documented here, may have fitness consequences for porpoises in densely trafficked areas. This hypothesis can be tested with onboard acoustic and multi-sensor tags where behavioural states, locomotion effort, feeding success, and ventilation rates can be logged in concert with noise exposure levels [e.g. 26].

We conclude that porpoises respond to low levels of medium- to high-frequency vessel noise. This finding is consistent with observations of ship avoidance at sea^34^, and points to a potentially large, but so far largely overlooked, conservation problem in areas of dense shipping and high porpoise numbers. The 63- and 125-Hz bands proposed in the European MSFD are not suited as measures of behavioural disturbance of porpoises whereas filtering using M-weighting^19^, loudness^35^ or the audiogram^37^ seem to provide a meaningful proxy for estimating behavioural disturbance with a tentative 50% onset at 123 dB re 1 μPa (rms, M-weighted) averaged over 30 s. Before implementation in mitigation measures and conservation efforts, we recommend that such a threshold should be tested thoroughly on a larger number of animals in the wild.

## Methods

### Data collection

The study took place between September 2011 and August 2012 at the Fjord&Belt, Kerteminde, Denmark, where four harbour porpoises are kept in a semi-natural net-pen complex. The enclosure (30 × 20 m^2^, average depth of 4 m) is situated in the canal connecting the Great Belt with Kerteminde Fjord, and is fenced off by a steel sheet piled wall alongshore, and nets on the two shorter ends.

Broadband recordings of vessels passing the enclosure were obtained with two calibrated Reson TC 4014 hydrophones (sensitivity: −186 ± 2 dB re 1 V/μPa between 0.01–160 kHz) that were placed at the opposite, open ends of the porpoise pen. Recordings at those locations were least subject to transmission loss, and thus precluded underestimation of noise levels received by animals within the pen complex, i.e. further from the source than the hydrophones. The hydrophones were connect to low-noise amplifiers: a Reson VP 2000 (3-pole band pass filter: 10 Hz–250 kHz) or a custom-built amplifier (1-pole high pass: 10 Hz, 4-pole low pass filter: 150 kHz) with 20, 30 or 40 dB gain. Signals were sampled at 0.5 or 1 MHz and saved as PCM*.wav files using 16-bit A/D converters (National Instruments USB-6251) controlled by programs written in LabVIEW (National Instruments). The self-noise of the recording system was measured in a silent and anechoic chamber at the Danish Technical University with the same configurations as used during the experiments. Different amplifiers and gain settings resulted in self-noise values varying by as much as 6 dB, therefore, only the maximum self-noise values were used.

Sound recording was started as soon as a boat came into view. Background ambient noise was recorded opportunistically when no boats were observed, but to minimise contribution of vessel noise in the ambient noise recordings, only audio files recorded 30 minutes before or 30 minutes after a vessel passage were later analysed. Hence, the number of background noise recordings selected for analyses varied from 2 to 6 per day, depending on the amount of vessel traffic.

Observations of porpoise behaviour were made simultaneously with vessel noise recordings. Response of the animals to boat presence was classified into two categories: “reaction” or “no reaction.” “Reaction” to noise was defined to occur when one or more animals suddenly and dramatically increased their swimming speed and sprayed the water upon surfacing in a stereotyped manner in a behaviour coined “porpoising” (see Supplementary Vid. S1). This type of behavioural response is commonly used in studies of noise influence on captive porpoises [e.g. 42,43]. “No reaction” response was defined as a lack of porpoising while the porpoises may have responded in other ways, inconsistent with the definition of porpoising.

### Ethics statement

The animals are maintained by Fjord&Belt under permits no. SN 343/FY-0014 from the Danish Ministry of Food, Agriculture and Fisheries, and 1996-3446-0021 from the Danish Forest and Nature Agency (under the Danish Ministry of the Environment). Their care and all experiments were approved by the IACUC committee of Aarhus University and are in strict accordance with the recommendations of the Danish Ministry of Food, Agriculture and Fisheries (issuing the permit to keep the animals), the Danish Ministry of the Environment (permit for catching the animals) and the Danish Council for Experiments on Animals.

### Data processing and signal analysis

A number of selection criteria were applied to the dataset in order to analyse the most representative levels of noise affecting the porpoises. For each vessel passage, only data from the station closest to the vessel were considered. Furthermore, recordings of boats that never got within 100 m of the station were excluded from further analysis. The selected files were then visually inspected using Adobe Audition 3.0 (Adobe Systems), to reject all recordings with clipped vessel noise or intense electrical noise.

Further sound analysis was custom-programmed in Matlab R2012b (MathWorks Inc.). All measurements were corrected for the frequency response of the hydrophones and the amplifiers.

Prior to further processing, relevant background and vessel noise sections of the audio files were extracted. For each background noise recording, the 30-second-long segment with the least broadband energy was identified visually using a spectrogram. For each vessel noise recording, a 2-minute-long segment with the most energy was selected. Within these segments, the 3-second- and 30-second-long time intervals with the highest energy contents were determined from bandpass filtered data using custom-written Matlab code. A bandpass filter with cut-off frequencies at 2–100 kHz (4^th^ order, Butterworth) was used to eliminate the contribution of electrical noise or wave noise (the transients resulting from wave actions on the pontoons), and exclude porpoise sounds from these fragments used to define the 3-second- and 30-second-windows. Sequences of 3 seconds before and 30 seconds around the time of harbour porpoise reaction were also selected to examine the noise recorded directly before the reaction was noted. Those fragments often differed in their levels from the ones with the most energy.

The selected segments of unfiltered noise were low-pass filtered at 100 kHz (4th order, Butterworth) to avoid the inclusion of omnipresent porpoise clicks in the level calculations. The broadband noise level was then quantified as root-mean-square sound pressure level over time windows of 3 seconds and 30 seconds. The four intervals (i.e., 3 seconds and 30 seconds with maximum energy vessel noise, and 3 seconds before and 30 seconds around the time of porpoise reaction) were used to explore the effects of averaging windows, differing in length and determination method, on rms measure of continuous, but varying noise^30^.

The detailed spectral features of the recorded noise were examined by means of power spectral density (PSD) analysis using the Welch’s method (8192 FFT points, 61- or 122-Hz bin width, non-overlapping rectangular window). The PSD levels were shown as normalised histograms of dB levels (histogram bin width of 1 dB) in 100-Hz frequency bins [based on 44]. The rms sound pressure levels were also computed in 36 third-octave bands (centre frequencies from 25 to 80000 Hz) according to ANSI standard S1.6-1984 using a filter bank (modified filtbank Matlab function provided by Christophe Couvreur, Faculte Polytechnique de Mons, Belgium). The third-octave bands were later combined into 12 octave bands (OL; centre frequencies from 31.5 to 63000 Hz) to simplify the number of variables fed to the GLMM.

Reported sensitivity of harbour porpoises to mid- and high-frequency impulsive sounds^45^,^46^ prompted us to test the effect of the echosounders at 200 kHz. Cumulative sound exposure levels (cSELs) were used as a proxy for accumulating received levels^47^ from all the pulses in a 30-second-long periods of high-pass filtered noise (Supplementary Fig. S1a). The echosounder pings were detected on 180-220 kHz passband filtered noise (4th order, Butterworth) using an automatic routine coded in Matlab. Only pulses above 114 dB re 1 μPa (pp) were selected for further analysis. Pulses lower than the set threshold were rarely detectable in 30 seconds with maximum energy vessel noise. Moreover they would have negligible contribution (<1 dB) to the cSELs which were dominated by the highest energy pings (see Supplementary Fig. S1a). The output of the automatic routine was verified manually in Matlab using a custom-made supervised detector. The sound exposure level (SEL) of each detected echosounder pulse was calculated from the high-pass filtered recordings (4th order, Butterworth, 160 kHz) in a time window containing 90% energy of the signal^30^,^47^.

Furthermore, following Southall *et al.*^19^ a marine mammal frequency weighted (M-weighted) rms sound pressure level was computed over the low-pass filtered (4th order, Butterworth, 100 kHz) vessel noise data. The M-weighting function is analogous to the C-weighting function from human audiometry and was here applied with the high-pass filter settings recommended by Southall *et al.*^19^ for functional hearing group of high-frequency cetaceans (i.e. with a cut-off frequency of 200 Hz).

### Statistical analysis

Statistical analysis was carried out using R statistical package version 2.15.2 (www.R-project.org). We performed a series of statistical tests to uncover the sound elements triggering the porpoise reaction to vessel noise. First, we tested for differences between four different averaging windows (i.e., 3 seconds and 30 seconds with maximum energy vessel noise, and 3 seconds before and 30 seconds around the time of porpoise reaction) using the Wilcoxon signed-rank test. Subsequently, the direct relationship between the broadband rms level and the probability of porpoise reaction, as well as the effect of the presence and level of echosounder pulses in the vessel noise, were assessed using GLMMs, where the occurrence of porpoise reaction to noise was the outcome. These three models were adjusted for the random effects of day, as a grouping factor, and the station, since for a given vessel only data from the station closest to the vessel were considered.

The relationship between the response of porpoises and received rms sound pressure level in different frequency bands (63- and 125-Hz third-octave bands, and 31.5–63000 Hz octave bands) was explored in a set of GLMMs with the presence of porpoise reaction to noise as an outcome. Due to the collinearity between third-octave and octave bands, a different model for each of the bands was used. Additionally, a more specific association between different octave band levels was studied by means of a biplot^32^. The initial dataset matrix consisted of the received rms sound pressure level in different frequency bands, with each row representing an individual recording (i.e. an observation), and each column containing a different frequency band (i.e. a variable). Once the matrix was centred by column and non-standardized, we performed a factorisation of it by means of singular value decomposition, and took the first two singular vectors to calculate the coordinates to create a two-dimensional biplot. In the biplot, each observation (individual noise recording) is represented as a point in a two-dimensional space and each variable (frequency band) as a vector. The spatial disposition of the observation points in a biplot shows the correlation structure of the observations in the two-dimensional space defined by the singular vectors. Therefore, a short distance between either two observations will indicate a stronger association. Similarly, the angle between vectors represents the correlation of the variables and the length of these vectors - the standard deviation of the variable. Consequently, the smaller the angle between two vectors, the stronger the correlation between the respective variables. This visual representation of the correlation structure of the data allowed us to find associations between either noise recordings or frequency bands, and combine octave-band levels into broader bandwidths. The resulting groups of correlated bands were subsequently used as individual explicative variables in the GLMMs. Another GLMM was performed to test the effect of the M-weighted rms sound pressure^19^. A directed acyclic graph was used to a priori identify the best possible strategy for potential confounders^48^. The GLMMs [63- and 125-Hz third-octave bands, and 31.5–63000 Hz octave bands, low- and high-frequency, M-weighted noise (rms)] were adjusted for the rms sound pressure level of the vessel noise, as it could potentially be related to the octave-band levels and hence influence the probability of reaction of the animals. The models were also adjusted for the random effects of the station and day.

The initial level of significance was 0.05. However, to prevent multiple comparison false discoveries in the GLMMs, the significance level of each test was re-evaluated with Benjamini-Hochberg-Yekutieli (BHY) procedure^49^ taking into account the 20 different models tested.

## Additional Information

**How to cite this article**: Dyndo, M. *et al.* Harbour porpoises react to low levels of high frequency vessel noise. *Sci. Rep.* **5**, 11083; doi: 10.1038/srep11083 (2015).

## Supplementary Material

Supplementary Information

Supplementary Video S1
