# Supplementary material for: Harbour porpoises react to low levels of high frequency vessel noise
Source: Sci Rep. 2015 Jun 22;5:11083. doi: 10.1038/srep11083 (PMC4476045; doi:10.1038/srep11083)
Supplement: Supplementary Information [file srep11083-s1.pdf]

Harbour porpoises react to low levels of high frequency vessel noise

Monika Dyndo, Danuta Maria Wiśniewska, Laia Rojano-Doñate, Peter Teglberg Madsen

Supplementary Information

The Supplementary Information includes one Supplementary Video, two Supplementary Figures and two Supplementary Tables.

Videos:

Supplementary Video S1. Harbour porpoises porpoising in response to noise from one of the boats passing by the Fjord&Belt facility.

<u>Figures S1-S2:</u>.

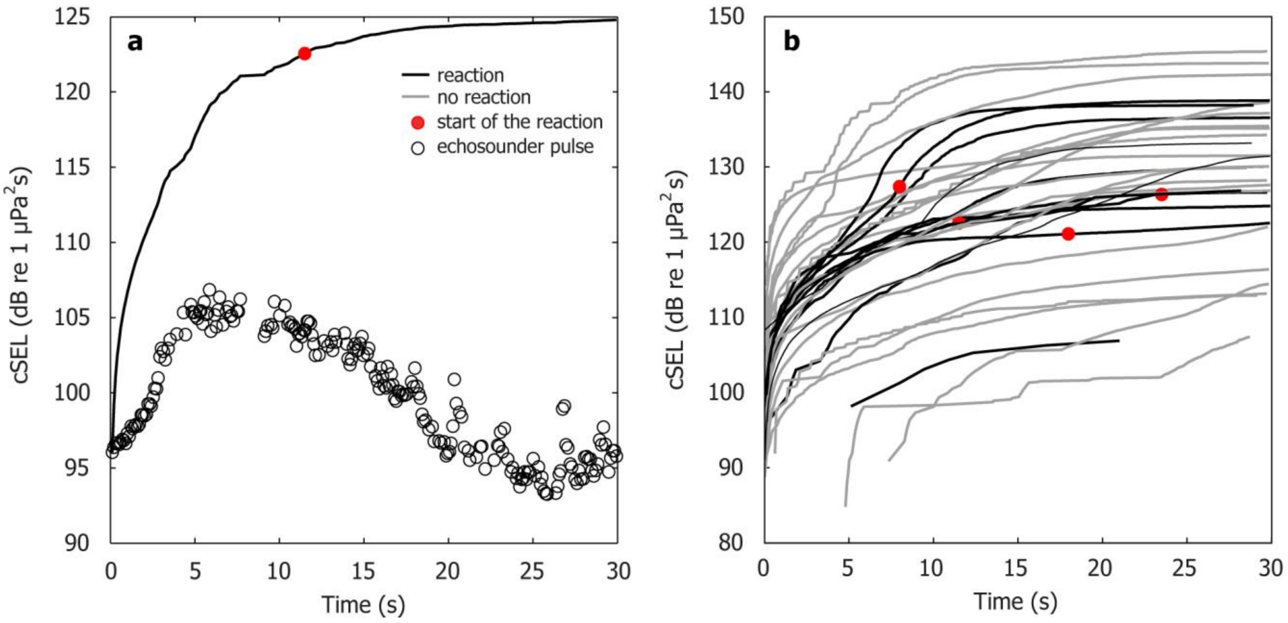

Supplementary Figure S1. Sound exposure levels (SELs) of 200-kHz echosounder pulses recorded at the porpoise enclosure during the passage of vessels that did (N=11) and did not (N=20) trigger a reaction. (a) An example showing the accumulation of energy (expressed as SEL) of the individual echosounder pings (black circles) over a 30-second time window with the highest energy in the echosounder frequency range (the highest energy segment was selected for 180-220 kHz pass-band recordings; the SEL were calculated for 160 kHz high-pass filtered recordings). This vessel did trigger a porpoising response and the time of reaction is indicated with a red circle marker. (b) Cumulative sound exposure levels (cSELs) of echosounder pulses from vessel that did (black lines) and did not (grey lines) elicit a distinct reaction of porpoises. The red dots mark time when porpoising was noticed. Please note that sometimes the reaction did not occur during the 30-second time window with the highest energy in the echosounder frequency range.

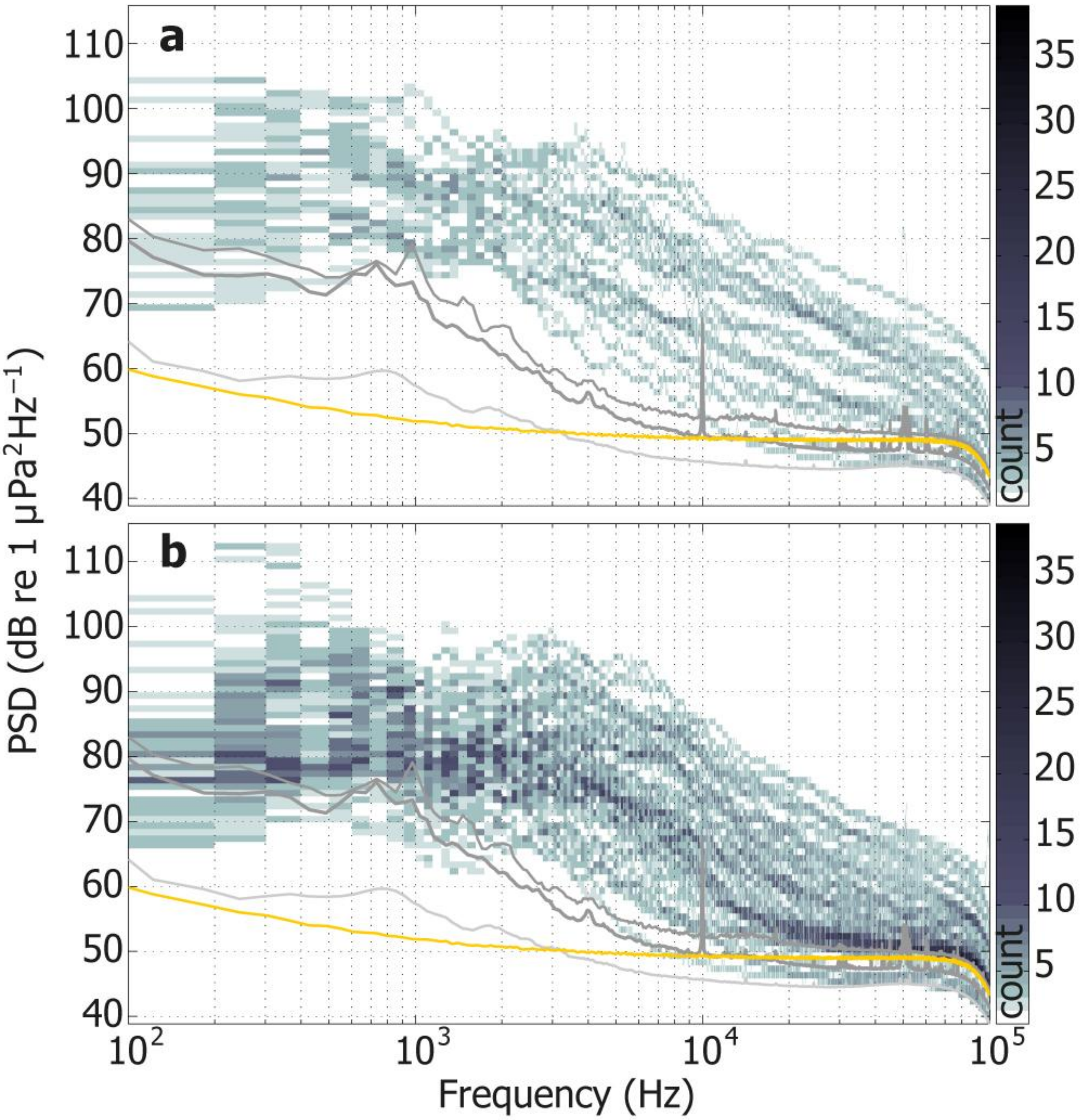

Supplementary Figure S2. Power Spectral Densities, PSD (8192 FFT points, 61 or 122 Hz bin width, non-overlapping rectangular window) of vessel noise (30 seconds with max. energy) that did (a) and did not (b) elicit porpoise reaction, shown as normalised histograms of dB levels (histogram bin width: 1 dB re 1 μPa^{2}Hz^{-1}) in 300 Hz frequency bins. Background noise PSD mean (light) and 10th and 90th percentiles (dark) are drawn as grey lines. The maximum possible self-noise PSD is indicated by the yellow line.

Tables S1-S2:

Supplementary Table S1. Results of different generalised linear mixed-effects models including the variables listed. Echosounder presence, echosounder cSEL and vessel noise rms pressure level were adjusted for the random effects of a day and station. TOL 63 and 125 Hz, OL 31.5 - 63000 Hz, low- and high- frequencies, M-weighted vessel noise (rms) were adjusted for the vessel noise rms sound pressure (averaged over 30 seconds with maximum energy), as well as the random effects of a day and station. P-value <0.05 is indicated by the '*'. Variables significant after Benjamini–Hochberg–Yekutieli correction (p-value$_{BHY}$ <0.05) are bolded. OR = odds ratio, CI = confidence interval, SD = standard deviation, cSEL = cumulative Sound Exposure Level, rms = root-mean-square, TOL = third octave level, OL = octave level.

variable	OR	95% CI	p-value	p-value$_{BHY}$	day variance (SD)	station variance (SD)
echosounder presence	2.6387	0.6162-11.3002	0.2236	0.9464	0.4367 (0.6609)	4.6298 (2.1517)
echosounder cSEL	1.0076	0.9972-1.0180	0.1476	0.8170	0.2487 (0.4987)	1.4731 (1.2137)
vessel noise rms pressure level	1.1167	0.9842-1.2670	0.1853	0.8414	0.7752 (0.8805)	3.8605 (1.9648)
TOL 63 Hz	1.1381	0.9763-1.3268	0.1683	0.8414	1.6930 (1.301)	8.7870 (2.964)
TOL 125 Hz	1.0172	0.9111-1.1357	0.7780	1.0000	0.7319 (0.856)	4.2154 (2.053)
OL 31.5 Hz	1.1267	0.9684-1.3110	0.1436	0.8170	1.3240 (1.151)	5.0470 (2.247)
OL 63 Hz	1.0998	0.9667-1.2511	0.2472	0.9882	1.8130 (1.347)	6.3260 (2.515)
OL 125 Hz	1.0343	0.9199-1.1630	0.6018	1.0000	0.7408 (0.861)	4.2849 (2.070)
OL 250 Hz	1.1052	1.0053-1.2150	0.0299*	0.1956	0.9179 (0.958)	3.3274 (1.824)
**OL 500 Hz**	**1.2474**	**1.0671-1.4582**	**0.0012***	**0.0276**	**0.1802 (0.425)**	**1.8757 (1.370)**
OL 1000 Hz	1.2541	1.0592-1.4849	0.0070*	0.0626	0.1402 (0.375)	0.4388 (0.662)
**OL 2000 Hz**	**1.2858**	**1.0863-1.5220**	**0.0029***	**0.0348**	**0.7166 (0.847)**	**0.0465 (0.216)**
OL 4000 Hz	1.1621	1.0148-1.3308	0.0162*	0.1164	1.1630 (1.079)	2.6460 (1.627)
OL 8000 Hz	1.1768	1.0311-1.3431	0.0067*	0.0626	1.5950 (1.263)	2.7190 (1.649)
**OL 16000 Hz**	**1.1926**	**1.0541-1.3493**	**0.0019***	**0.0331**	**1.5750 (1.255)**	**3.8420 (1.960)**
**OL 31500 Hz**	**1.1824**	**1.0544-1.3258**	**0.0023***	**0.0331**	**1.2860 (1.134)**	**3.8170 (1.954)**
OL 63000 Hz	1.1962	1.0337-1.3842	0.0141*	0.1129	1.2370 (1.112)	4.3220 (2.079)
low frequencies (22.4 - 178 Hz)	1.1145	0.9615-1.2919	0.1871	0.8414	1.3690 (1.170)	5.0260 (2.242)
**high frequencies (0.2 - 89 kHz)**	**1.3712**	**1.0860-1.7314**	**0.0008***	**0.0273**	**0.5564 (0.746)**	**0.1164 (0.341)**
**M-weighted vessel noise (rms)**	**1.4124**	**1.1200-1.7811**	**0.0004***	**0.0273**	**0.4233 (0.651)**	**0.0003 (0.017)**

Supplementary Table S2. Parameters of the recorded vessel noise calculated over 30-second-long segments with maximum energy. SD - standard deviation, IQR - interquartile range, cSEL = cumulative Sound Exposure Level, rms = root-mean-square, TOL = third octave level, OL = octave level.

variable	mean (SD)	median (IQR)
echosounder cSEL [dB re 1μPa^{2}s]	129 (10)	130 (125 - 135)
vessel noise rms pressure level [dB re 1μPa]	130 (11)	138 (119 - 140)
TOL 63 Hz [dB re 1μPa]	91 (6)	92 (89 - 94)
TOL 125 Hz [dB re 1μPa]	88 (6)	89 (86 - 92)
OL 31.5 Hz [dB re 1μPa]	100 (6)	100 (97 - 103)
OL 63 Hz [dB re 1μPa]	97 (7)	97 (93 - 99)
OL 125 Hz [dB re 1μPa]	94 (6)	95 (92- 98)
OL 250 Hz [dB re 1μPa]	107 (10)	107 (100 - 112)
OL 500 Hz [dB re 1μPa]	116 (9)	114 (108 - 123)
OL 1000 Hz [dB re 1μPa]	116 (7)	116 (111 - 121)
OL 2000 Hz [dB re 1μPa]	117 (7)	117 (111 - 122)
OL 4000 Hz [dB re 1μPa]	116 (9)	115 (111 - 123)
OL 8000 Hz [dB re 1μPa]	112 (10)	111 (106 - 120)
OL 16000 Hz [dB re 1μPa]	107 (9)	107 (100 - 115)
OL 31500 Hz [dB re 1μPa]	104 (8)	102 (99 - 111)
OL 63000 Hz [dB re 1μPa]	103 (6)	102 (100 - 107)
low frequencies (31.5 - 125 Hz) [dB re 1μPa]	108 (6)	103 (100 - 106)
high frequencies (0.25 - 63 kHz) [dB re 1μPa]	130 (7)	123 (118 - 130)
M-weighted vessel noise (rms) [dB re 1μPa]	121 (7)	120 (114 - 127)
